# Pure Laparoscopic Versus Hand-Assisted Nephrectomy: Comparative Outcomes in Living Kidney Donors

**DOI:** 10.7759/cureus.79191

**Published:** 2025-02-17

**Authors:** Nelson Rosario Almonte, Antonio Esqueda-Mendoza, Manuel E Mendoza Arcila, Hector Rendon Dosal, Norma Z Marique Canche

**Affiliations:** 1 Urology Department, Hospital Regional de Alta Especialidad de la Peninsula de Yucatan del IMSS Bienestar, Merida, MEX; 2 Urology Department, Hospital Star Médica de Merida, Merida, MEX; 3 Trasplant Surgery Department, Hospital Regional de Alta Especialidad de la Peninsula de Yucatan del IMSS Bienestar, Merida, MEX; 4 Urology Department, Universidad Autónoma de Yucatán, Merida, MEX

**Keywords:** hand-assisted, kidney transplantation, laparoscopic donor nephrectomy, living donor nephrectomy, living donor renal transplant

## Abstract

Introduction: Kidney transplantation remains the only curative therapy for end-stage renal disease, with living donor nephrectomy playing a vital role in addressing the shortage of deceased donors. This study compares the effectiveness and safety of two minimally invasive surgical techniques for kidney graft procurement: pure laparoscopic nephrectomy and hand-assisted laparoscopic nephrectomy.

Materials and methods: This retrospective, observational study analyzed 50 living donor nephrectomies performed between 2021 and 2024 at a high-specialty hospital in Mexico. Sociodemographic, clinical, and surgical variables - including surgical time, bleeding, warm ischemia time, complications (Clavien-Dindo classification), and creatinine levels - were evaluated. Statistical analyses were conducted to assess differences between the two techniques.

Results: The hand-assisted technique was performed in 31 (62%) cases, while the pure laparoscopic approach was used in 19 (38%). The mean surgical time (203.54 ± 62.72 min) and bleeding volume (258.78 ± 536.67 mL) showed no statistically significant differences between the techniques (p > 0.05). Complications occurred in four (8%) patients, with higher-grade complications (3B and 4B) observed exclusively in the pure laparoscopic group. Creatinine levels demonstrated no significant differences between groups at any postoperative interval.

Discussion: Both techniques proved to be safe and effective for living donor nephrectomy, with comparable outcomes in surgical parameters and donor renal function. The hand-assisted technique offered advantages in terms of lower bleeding variability, while pure laparoscopic nephrectomy was associated with higher-grade complications in isolated cases.

Conclusions: Living donor nephrectomy is a safe and effective procedure using either technique, emphasizing the importance of institutional expertise and individualized surgical planning. Future research should focus on multicenter studies, donor quality of life, and the integration of advanced technologies such as robotic systems to optimize outcomes.

## Introduction

Kidney transplantation is the definitive treatment for end-stage renal disease (ESRD), offering superior outcomes compared to renal replacement therapies. The increasing demand for kidney transplantation arises from the persistent shortage of deceased donors, which remains a global challenge. In Mexico, 16,629 patients were on the kidney transplant waiting list in the first quarter of 2024, with only 3,069 transplants performed in 2023; 2,164 of these were from living donors, who provide grafts with the highest survival rates [1-3].

Although kidney transplantation is known to be the ideal treatment over renal replacement therapy, the scarcity of deceased donors has posed a challenge. However, the response to this situation has been an increase in the availability of living donors. This is also complemented by the fact that living donors provide grafts with the highest survival rates [2].

Since its introduction in 1995 [2-4], laparoscopic living donor nephrectomy has become the gold standard for kidney graft retrieval due to its minimally invasive nature, reduced postoperative pain, and faster recovery. The left kidney is often preferred for donation because of its elongated renal vein, facilitating surgical manipulation and implantation [2-4].

To date, multiple variants have been developed for the laparoscopic technique, supported by advancements in minimally invasive technologies. These range from hand-assisted laparoscopic nephrectomy, single-port laparoscopic techniques, retroperitoneoscopic approaches, laparoendoscopic methods, and robotic-assisted laparoscopic renal graft procurement [4]. Each of these techniques has its benefits and challenges, and their selection must consider familiarity with the technique, costs, availability, and expertise of the surgical staff [5,6].

This study evaluates the surgical outcomes and safety of procedures for kidney graft procurement. Currently, this hospital performs two techniques: the pure laparoscopic technique and the hand-assisted technique. The surgeon's preference and experience play a major role in deciding between techniques. Despite the fact that both methods are well-established, surgeons typically choose the one with which they are most familiar, which can affect the effectiveness and results of operations. Both techniques are always performed on living donors. We currently do not have a deceased donor program, nor do we perform open or robotic-assisted procedures.

The surgical techniques used in this study follow well-established laparoscopic protocols for living donor nephrectomy, ensuring safety and reproducibility. In the pure laparoscopic approach, transperitoneal access is created with three 12 mm trocars. (A lower quadrant 12-mm trocar is placed approximately two fingerbreadths medial and superior to the anterior superior iliac spine. Either a second 12-mm trocar is placed in the midclavicular line approximately 2 cm below the costal margin. The camera trocar, also a 12-mm trocar, is placed superior and lateral to the umbilicus and situated between the two working trocars.) The kidney is dissected using LigaSure. The renal artery and vein are carefully isolated, clipped proximally and distally with Hem-o-Lock clips, and divided with cold scissors to minimize thermal injury. The ureter is divided at the level of the iliac vessels, and the kidney is retrieved through an extended trocar incision using a GelPort system. In the hand-assisted technique, an initial supraumbilical incision allows for early manual assistance via a GelPort substituting the left working 12-mm trocar, facilitating tissue handling and vascular dissection while maintaining pneumoperitoneum. Both techniques involve meticulous hemostasis, retroperitoneal dissection along Toldt’s fascia, and standard laparoscopic closure. These standardized approaches ensure comparability and reproducibility across surgical teams and institutions.

Although minimally invasive procedures for living donor nephrectomy have become widely used, there is still disagreement on the best course of action, especially in areas with limited resources. This study is necessary to provide comparative evidence on the safety and efficacy of pure laparoscopic versus hand-assisted techniques, offering valuable insights into their clinical applicability. Our research aims to improve donor nephrectomy decision-making and modify surgical protocols by assessing important surgical and functional outcomes.

## Materials and methods

Study characteristics 

This retrospective, cross-sectional, observational study analyzed cases of laparoscopic living donor nephrectomies performed between 2021 and 2024 at the Regional High Specialty Hospital of the Yucatan Peninsula IMSS Bienestar in Mérida, Mexico. Clinical and surgical data were extracted from patient records. Informed consent was obtained from all donors, who were informed of the risks and benefits of the procedure and the use of their anonymized data for research.

Study variables 

Collected variables included age, sex, laterality of the donated kidney, body mass index (BMI), surgical approach, surgeon, operative time (minutes), estimated blood loss, need for conversion to open surgery, warm ischemia time, complications (graded using the Clavien-Dindo classification), and creatinine levels (preoperative and postoperative). The choice of surgical technique (pure laparoscopic or hand-assisted) was determined by the operating surgeon during preoperative planning. Patients with metabolic comorbidities were excluded from donation.

Inclusion and exclusion criteria 

All patients who underwent laparoscopic living-donor nephrectomies and had given their written consent were included. Patients whose records were incomplete were excluded. Between 2021 and 2024, 62 laparoscopic living donor nephrectomies were performed, of which 50 were included; the 12 remaining patients were excluded due to incomplete data. The sample chosen for the present study was carried out using non-probabilistic convenience sampling; this is due to the fact that the investigators desired to obtain as large a sample as possible in the period of data collection.

Statistical processing 

Data collected were entered into Microsoft Excel (Microsoft® Corp., Redmond, WA) and analyzed using Statistical Product and Service Solutions (SPSS, version 25; IBM SPSS Statistics for Windows, Armonk, NY). Statistical tables and tests were generated for analysis.

Ethical considerations 

This study is a retrospective, descriptive analysis utilizing pre-existing data from patient medical records. All included patients had previously signed informed consent forms authorizing the use of their clinical data for research purposes. The data were fully anonymized to ensure the confidentiality and privacy of the participants, preventing any possibility of identification.

Given that the study did not involve direct interaction with patients, posed a minimal risk, and adhered to ethical standards for research with anonymized data, institutional guidelines determined that formal submission to an ethics committee was not required. However, the principles outlined in the Declaration of Helsinki and relevant national and institutional regulations were strictly followed throughout the research process.

## Results

The sociodemographic and general data of the patients are summarized in Table 1. A total of 62 patients underwent living donor nephrectomy, of which 50 (100%) were included in this study. Among these, there was a predominance of women (28, 56%) over men (22, 44%). The mean age was 34.34 ± 11.23 years. The average BMI was 27.35 ± 3.47, and 11 (22%) patients were obese (BMI ≥ 30).

**Table 1:** Sociodemographic and body weight characteristics of the study population (N=50)

Variable	Number	Percentage	Media ± DS
Age (years)			34.34 ± 11.23
Male	22	44%	
Female	28	56%	
Body mass index			27.35 ± 3.47
(Obesity BMI ≥30)	11	22%	

Table 2 shows that most patients donated the left kidney (46, 92.0%). Regarding the distribution of approaches, they were as follows: hand-assisted: 31 (62.0%) and pure laparoscopic: 19 (38.0%). The mean time for warm ischemia was 2.98 ± 2.08 minutes.

**Table 2:** Distribution of kidney laterality, surgical approach, and warm ischemia time (N=50)

Variable	Number	Percentage	Average ± SD
Left	46	92%	
Right	4	8%	
Hand-assisted	31	62%	
Pure laparoscopic	19	38%	
Warm ischemia time			2.98 ±2.08 min

Table 3 displays that the overall mean bleeding was 258.78 ± 536.67 mL. In the pure laparoscopic group, the mean bleeding was 421.05 ± 834.18 mL, while in the hand-assisted group, it was 156 ± 128.94 mL. The p-value for bleeding comparison was 0.186, indicating no statistically significant difference.

**Table 3:** Statistical comparison of bleeding and surgical time between pure laparoscopic and hand-assisted living donor nephrectomy techniques, including mean values and p-values Using an independent sample t-test.* Bleeding t-statistic value: 1.38, with a standard error of 192.6. For surgical time t-statistic value: -1.39, with a standard error of 17.66.

Variable	Media ± DS	Pure Laparoscopic	Hand Assisted	P-value
Bleeding	258.78 mL ± 536.67 mL	421.05 mL ± 834.18 mL	156 mL ± 128.94 mL	0.186*
Surgical Time	203.54 min ± 62.72 min	188.12 min ± 58.14 min	212.59 min ± 64 .51	0.194*

The overall mean surgical time was 203.54 ± 62.72 minutes. For the pure laparoscopic group, the mean time was 188.12 ± 58.14 minutes, and for the hand-assisted group, it was 212.59 ± 64.51 minutes. The p-value for surgical time comparison was 0.194, indicating no statistically significant difference.

The statistical analysis of bleeding and surgical time between the Pure Laparoscopic and Hand-Assisted groups was conducted using an independent sample t-test. For bleeding, the calculated t-statistic value was 1.38, with a standard error of 192.6, reflecting no statistically significant difference between the groups (p=0.186). Similarly, for surgical time, the t-statistic value was -1.39, with a standard error of 17.66, indicating no statistically significant difference (p = 0.194). These findings suggest that, while differences in means were observed, the variability within the groups precludes drawing conclusions of statistical significance.

Table 4 shows that four (8%) of the patients presented complications, distributed as follows: one (2%) patient who presented fever for a grade 1, one (2%) who presented lesser vascular injury that required postoperative revision with exploratory laparotomy for a grade 3B, and two (8%) 4B vascular injuries of the renal vein in both cases that required conversion to an open technique. All high-grade complications (3B and 4B) were observed in the pure laparoscopic group. A chi-square test revealed no statistically significant difference in complication rates between groups (p = 0.293).

**Table 4:** Distribution of complication degrees according to the Clavien-Dindo classification in pure laparoscopic and hand-assisted living donor nephrectomies Statistical chi-square test*. Chi-square results: X2=1.11 p-value: 0.293

Degree of Complication	Pure Laparoscopic	Hand Assisted	P-value
1		1	
3B	1		
4B	2		
			0.293*

Regarding the progression of global creatinine, the creatinine levels were monitored at different times as a predictor of postsurgical safety, as summarized in Table 5.

**Table 5:** Progression of global creatinine levels

Time Period	Mean Creatinine (mg/dL)	Standard Deviation (mg/dL)
Preoperative	0.79	0.18
First 90 Days	1.31	0.89
6 Months	1.06	0.24
12 Months	0.97	0.28

Regarding the progression of creatinine levels measured by surgical approach groups, Table 6 presents a comparative analysis of creatinine levels between the pure laparoscopic and hand-assisted surgical approaches at various postoperative intervals. Preoperative creatinine levels were similar between groups (0.77 ± 0.16 mg/dL vs. 0.80 ± 0.19 mg/dL, p = 0.537). At 90 days, both groups exhibited an increase, with a higher mean in the pure laparoscopic group (1.49 ± 1.39 mg/dL) compared to the hand-assisted group (1.19 ± 0.28 mg/dL), although this difference was not statistically significant (p = 0.376). By six months, creatinine levels had stabilized and were comparable between the groups (1.00 ± 0.27 mg/dL vs. 1.09 ± 0.24 mg/dL, p = 0.534). At 12 months, levels approached baseline, with the pure laparoscopic group showing slightly lower creatinine levels (0.79 ± 0.16 mg/dL) compared to the hand-assisted group (1.06 ± 0.29 mg/dL); however, this difference did not reach statistical significance (p = 0.123). Overall, no statistically significant differences were observed between the groups at any time point.

**Table 6:** Comparison of creatinine levels over time between pure laparoscopic and hand-assisted groups T-test was used. T-statistic values for creatinine level comparisons at each postoperative time point were as follows: -0.22 (presurgical), 1.16 (first 90 days), -1.36 (6 months), and -4.46 (12 months).

Variable	Presurgical	First 90 days	6 months	12 months
Pure laparoscopic	0.79 mg/dL ± 0.16	1.49 mg/dL ± 1.39	1 mg/dL ± 0.27	0.79 mg/dL ± 0.16
Hand-assisted	0.80 mg/dL ± 0.19	1.19 mg/dL ± 0.28	1.09 mg/dL ± 0.24	1.06 ± 0.29
P-value*	p=0.537	p=0.376	p=0.534	p=0.123

A comparison was made between blood loss, the presence of complications, and the degree of complications (Clavien-Dindo). In Tables 7-8, the analysis of blood loss in living donor nephrectomies revealed significant variations based on the presence and degree of complications. Among the 50 cases, the majority, 46 (92%) experienced no complications, with an average blood loss of 128.44 ml ± 99.27 mL. In contrast, in cases with complications, four (8%) demonstrated a markedly higher average blood loss of 1,725 mL (± 1,158.66 mL). When stratified by the Clavien-Dindo classification, patients without complications (grade 0, 46=92%) again exhibited the lowest average bleeding at 128.44 mL (± 99.27 mL). Cases with minor complications (grade 1, 1=2%) had an average blood loss of 500 mL, while those with more severe complications (grade 3B, 1=2%) showed an average of 1,200 mL. The highest blood loss was observed in patients with grade 4B complications (2=4%), averaging 2,600 mL (± 848.53 mL). These findings highlight the critical association between the severity of complications and the extent of blood loss in this surgical cohort.

**Table 7:** Bleeding volume in living donor nephrectomies categorized by the presence or absence of complications

Complication	Number	Percentage	Average Bleeding
No	46	92%	128.44 mL ± 99.27 mL
Yes	4	8%	1725 mL ±1158. 66 mL

**Table 8:** Distribution and average bleeding volume by degree of complication according to the Clavien-Dindo classification

Degree of Complication	Number	Percentage	Average Bleeding
0 (No)	46	92%	128.44 mL ± 99.27 mL
1	1	2%	500 mL
3B	1	2%	1200 mL
4B	2	4%	2600 mL ± 848.53 mL

## Discussion

Kidney transplantation remains a cornerstone in medicine, transforming chronic kidney disease (CKD) into a curable condition. However, the procurement of kidney grafts from living donors remains a significant surgical challenge. This procedure requires careful donor evaluation to ensure a viable graft, expert surgical skills, and measures to minimize any impact on the donor's quality of life. Despite these precautions, the procedure is not without risks and complications.

In this study, the sociodemographic and general variables were consistent with findings reported in the literature regarding sex and age distribution. Women accounted for 28 (56%) of the population, and the mean age was 34.34 ± 11.23 years. The average BMI was 27.35 ± 3.47, aligning with values reported in previous studies. Notably, 11 (22%) of the patients were obese (BMI ≥ 30), a variable not frequently examined in comparable studies. Authors such as Serrano-Ardila et al. [5] suggested that obesity was historically considered a risk factor for complications or renal function deterioration. However, in our series, obesity was not associated with complications or prolonged warm ischemia time.

In alignment with the literature, most patients, 46 (92%) in this study underwent left nephrectomy. This preference is attributed to the technical ease of the left-sided approach, which avoids liver manipulation and provides longer renal veins. Right nephrectomies were performed in cases where preserving the donor’s optimal renal unit or addressing minor vascular anomalies was prioritized. Regarding the surgical approach, 31 (62%) of patients underwent hand-assisted nephrectomy, as determined by the surgeon’s preference. Warm ischemia time, a critical predictor of graft function, was consistent with previously reported findings, regardless of the surgical technique used [7].

Surgical time and bleeding are factors in considering the safety of transplantation. When comparing the results in the group of hand-assisted surgery patients with the group of Wiborg et al. [7], we obtained a shorter surgery and bleeding time, but in very similar ranges. However, when comparing the pure laparoscopy group with other authors, the volume of blood loss was higher, this was associated with the presence of high-grade complications (Clavien-Dindo grades 3 and 4) that did not represent a statistical significance. In this regard, when compared with other mixed series, the presence of complications, despite being of a high degree, occurred in a small proportion and is similar to the literature reviewed for this study [5,8,9].

Complications were observed in four (8%) patients in this series, ranging from grade 1 to grade 4B. All cases were resolved successfully. This complication rate is comparable to other studies [2,5,8], where vascular complications similarly predominated. Although a slight association was noted between complications and increased bleeding, it did not reach statistical significance due to the low frequency of these events. As expected, higher complication grades correlated with greater average blood loss.

Residual renal function was assessed using creatinine as a parameter. Unlike most reviewed studies [5,7,8], which only reported postoperative creatinine levels, our study monitored creatinine at four intervals: preoperative, 90 days, six months, and 12 months. The findings were comparable to those reported in the literature [6].

Wang et al. [10] conducted a meta-analysis comparing laparoscopic donor nephrectomy (LDN) and open donor nephrectomy (ODN), reporting no significant differences in long-term outcomes, including serum creatinine levels, hypertension, proteinuria, and graft survival. Our study, focusing exclusively on laparoscopic techniques in living donors, supports these findings regarding the safety and efficacy of LDN in maintaining kidney function and donor health. Additionally, Wang et al. highlighted the advantages of LDN, such as reduced pain and faster recovery, which align with our findings of favorable postoperative recovery and donor satisfaction. These results underscore the need to refine minimally invasive techniques to improve donor and recipient outcomes [10].

Broudeur et al. [11] systematically reviewed the feasibility and safety of laparoscopic living donor nephrectomy (LLDN) in complex cases, such as right kidney and multiple-renal artery nephrectomies. They found that, despite the increased technical complexity, outcomes such as graft loss, delayed graft function, and donor safety metrics were comparable to standard procedures. In contrast, our study focuses on left kidney nephrectomies, emphasizing the advantages of this approach in terms of surgical simplicity and donor safety. Both studies highlight the importance of thorough preoperative planning and technique selection to optimize outcomes. While Broudeur et al. demonstrated the versatility of LLDN in challenging scenarios, our findings reinforce its safety and efficiency in routine applications [11].

Wang et al. [12] conducted a meta-analysis comparing robotic-assisted donor nephrectomy (RDN) with LDN, revealing that LDN is associated with shorter operative times, shorter warm ischemia times, and reduced estimated blood loss. These findings align with the present study, which focuses exclusively on LDN and highlights its efficiency and safety as a preferred approach for living donor nephrectomy. They also noted that RDN may reduce postoperative pain due to less tissue trauma at the port sites, a feature not explored in this study but worth considering for future research. Both studies emphasize the importance of refining techniques to enhance donor safety and surgical outcomes, with our work providing additional insight into optimizing laparoscopic approaches [12].

When comparing this study with that of Hernández-Rivera et al. [13] both studies explore the functionality and outcomes of kidney transplants, focusing on risk factors and one-year results. While Hernández-Rivera et al. include transplants from both living and deceased donors, this study exclusively focuses on living donors, thereby avoiding complications associated with deceased donors, such as prolonged cold ischemia times. The reported complication rate of 8% in this study aligns with the severe complication rates in Hernández-Rivera's work, where infections and rejection were major contributors. This methodological difference underscores the safety of procedures in living donors, highlighting our contribution to mitigating risks within a specific population [13].

Windisch et al. [14] compared RDN with hand-assisted laparoscopic donor nephrectomy (HLDN) and identified significant differences in operative time, learning curves, and postoperative recovery. Their findings revealed that RDN required longer operative times (287 vs. 160 minutes, p < 0.01) but offered shorter hospital stays (3.9 vs. 5.7 days, p < 0.01), while warm ischemia times were comparable between the two techniques. Similarly, this study highlights the safety and efficiency advantages of laparoscopic nephrectomy. However, while we focus solely on laparoscopic approaches, Windisch et al. provided valuable insights into the potential benefits of robotic assistance, particularly in enhancing maneuverability and reducing the learning curve. These findings align with our results regarding the importance of optimizing surgical techniques to balance efficiency and donor safety [14].

This study has limitations that should be considered when interpreting its findings. First, its retrospective design and observational nature inherently carry a risk of bias in data collection and analysis, potentially limiting the generalizability of the results. Additionally, the sample size, while sufficient for a single-center study, may lack the statistical power required to detect significant differences between surgical techniques, particularly in variables with high variability, such as blood loss. Lastly, the absence of a control group, including open or robot-assisted procedures, restricts broader comparisons and makes it challenging to position these techniques within the full spectrum of available surgical approaches.

## Conclusions

In kidney transplantation, living donor nephrectomy remains a critical procedure that ensures donor safety while providing high-quality grafts. This study demonstrates that both hand-assisted and pure laparoscopic techniques are safe and effective minimally invasive approaches for kidney procurement. Although no statistically significant differences were observed in surgical time, bleeding, or renal function outcomes, the hand-assisted technique showed a trend toward fewer high-grade complications, making it a slightly preferable option in certain clinical scenarios.

The findings underscore the importance of tailoring the surgical approach based on patient characteristics, institutional expertise, and available resources. Specifically, the hand-assisted method, with its lower variability in blood loss and reduced incidence of severe complications, may be particularly advantageous for less experienced surgeons or in cases where technical challenges are anticipated. Future research should focus on multicenter, long-term studies to further evaluate the impact of these techniques on donor quality of life and recipient outcomes. By continuing to refine these surgical approaches, the medical community can enhance both donor and recipient outcomes, ensuring the sustainability and success of kidney transplantation programs.
